# An Evolutionary Perspective of Nutrition and Inflammation as Mechanisms of Cardiovascular Disease

**DOI:** 10.1155/2015/179791

**Published:** 2015-11-29

**Authors:** María Esther Rubio-Ruiz, Ana Elena Peredo-Escárcega, Agustina Cano-Martínez, Verónica Guarner-Lans

**Affiliations:** Department of Physiology, Instituto Nacional de Cardiología “Ignacio Chávez”, 14080 Mexico City, DF, Mexico

## Abstract

When cardiovascular diseases are viewed from an evolutionary biology perspective, a heightened thrifty and an inflammatory design could be their mechanisms. Human ancestors confronted a greater infectious load and were subjected to the selection for proinflammatory genes and a strong inflammatory function. Ancestors also faced starvation periods that pressed for a thrifty genotype which caused fat accumulation. The pressure of sustaining gluconeogenesis during periods of poor nourishment selected individuals with insulin resistance. Obesity induces a proinflammatory state due to the secretion of adipokines which underlie cardiometabolic diseases. Our actual lifestyle needs no more of such proinflammatory and thrifty genotypes and these ancestral genes might increase predisposition to diseases. Risk factors for atherosclerosis and diabetes are based on inflammatory and genetic foundations that can be accounted for by excess fat. Longevity has also increased in recent times and is related to a proinflammatory response with cardiovascular consequences. If human ancestral lifestyle could be recovered by increasing exercise and adapting a calorie restriction diet, obesity would decrease and the effects on chronic low-grade inflammation would be limited. Thereby, the rates of both atherosclerosis and diabetes could be reduced.

## 1. Introduction

The primary causes of death during most of the history of humanity included infection and famine. Therefore, evolutionary pressures during the Miocene and Pleistocene periods selected for individuals with large portions of the genome dedicated to inflammatory responses and innate immunity that are able to counteract infection and allow for survival to trauma. It also selected genes involved in the accumulation of nutrient stores favoring individuals who were able to perform gluconeogenesis and develop insulin resistance, promoting a thrifty genotype with accumulation of fat deposits. Atherosclerosis and diabetes, which are actually among the main causes of death in humans, were absent in our hunter-gatherer ancestors whose lifestyle was characterized by extended periods of physical activity and a high-protein diet [1]. In addition to the protection from periods of food scarcity, fat stores constitute the origin of the energy needed to synthesize acute phase proteins which form part of the inflammatory responses, therefore connecting nutrition and inflammation [2, 3].

Apparently, the resulting genetic design of human evolutionary pressures that our ancestors underwent went hand in hand with an active lifestyle and a diet including calorie restriction. This design responds and adapts to a previous lifestyle which is not our current way of living dominated by increasingly sedentary habits, an abundance of foods rich in carbohydrates, and a diminished risk of mortality from common infections due to the strengthening of the immune system or by the treatment with antibiotics [1, 4, 5].

Inflammatory responses and metabolic imbalances are risk factors associated with cardiovascular disease and diabetes [6]. The fact that diabetic patients are more prone to premature atherosclerosis has led to the hypothesis that both conditions have been favored by evolutionary pressures and may have a common genetic and inflammatory basis [6]. As a result of our thrifty genotype, we developed a low-degree chronic and systemic inflammatory state, promoted by the secretion of proinflammatory cytokines by adipocytes. Furthermore, alterations in the innate immune system due to our proinflammatory genotype are linked to insulin resistance, diabetes, and cardiovascular disease [7–10]. Adipose tissue releases adipokines such as leptin and acute phase reactants, which generate inflammation, decrease immune responses, and increase susceptibility to infections.

The lifespan of the human population has also increased in recent years. In women, a state of chronic inflammation, accompanied by oxidative stress and decreased ovarian function, is observed, while in men, oxidative stress is accompanied by a decrease in the production of proinflammatory cytokines as in glucocorticoid induced stress [11]. In healthy elderly individuals, the hypothalamus-pituitary-adrenal axis (HPA) is activated in an unbalanced way, resulting in enhanced stress when compared to that present in the young population. Immunological changes during aging are similar to those observed after chronic stress or treatment with glucocorticoids and at the cellular level they are known as immunosenescence [12]. This condition could also contribute to metabolic syndrome (MS) and diabetes and their cardiovascular consequences.

During aging, lapses of inflammatory stimulus act continually as challenges to destabilize homeostasis. Therefore, aging is considered as a process of constant remodeling. This proposal complies with the hypothesis that the presence of favorable genetic variants for the survival during the reproductive ages and/or adapted to an ancestral proinflammatory environment may become detrimental in the postreproductive age which is further accentuated in the elderly [13].

**Figure 1:**
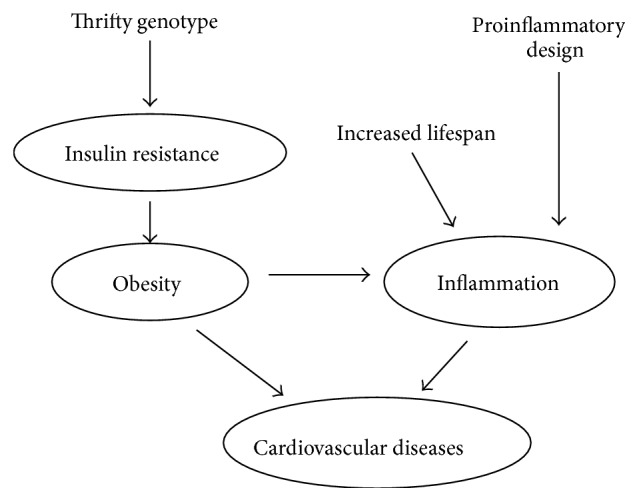
Evolutionary traits that lead to insulin resistance, obesity, and cardiovascular diseases.

In this paper, we discuss the possibility that the current epidemic of diabetes and atherosclerosis which is being faced by the human population could have been predicted from the point of view of the principles of biological evolution in which humans are poorly adapted to the current lifestyle. This could be assumed based on the adaptation acquired by our ancestors to an energetically demanding environment with moderate access to food. This adaptive pattern is no longer compatible with our actual living environment in which demand for energy expenditure is limited and there is immediate access to copious food. The main situations to which we are currently not well adapted are summarized in Table 1 and traits that lead to insulin resistance, obesity, and cardiovascular diseases are shown in Figure 1.

## 2. Evolution of Human Nutrition

Six million years ago, human primate ancestors lived in the woods of Eastern Africa and their diet was comprised of leaves, roots, fruits, and nuts [14, 15]. The main macronutrients consumed were carbohydrates having low glycemic index and individuals exerted high physical activity. The climate became dyer and colder and the forests in which our ancestors lived disappeared and were replaced by arid grassland. This change forced the human ancestors to move to the coastlines. The human diet changed to low carbohydrate, high-protein and became rich in micronutrients such as iron, retinol, zinc, vitamin B_12_, and unsaturated fatty acids from fish (with a balance of fatty acids omega-3 and omega-6, ratio 1 : 1), as hunted large predators became part of the diet. These new components of the diet provided enough fuel and building blocks to facilitate encephalization and the development of intellectual capacity [14, 16, 17].

A theory of the “selfish brain” which may play a central role in obesity has been proposed and it might have been a determinant in the evolution of insulin resistance. 50% of the total body glucose is consumed by the brain even if it constitutes only 2% of the body mass and the brain depends exclusively on glucose as its energy source consuming more than 20% of total resting expenditure [14]. The brain is able to maintain a constant flux of large amounts of glucose in the presence of powerful competitors such as fat and muscle tissue by activating a stress system that includes the HPA axis and the sympathetic nervous system. Activation of the sympathetic-adrenal system inhibits glucose uptake by peripheral tissues by inhibiting insulin release and inducing insulin resistance and by increasing hepatic glucose production [18]. Furthermore, insulin signals the brain so that it is the first organ to replenish its source of glucose before other organs consume it [19]. Gastrointestinal hormones such as leptin may represent an evolutionary adaptation. Leptin crosses the blood brain barrier informing the brain about the feeding and nutritional status of the rest of the body and hypertriglyceridemia which occurs during starvation inhibits leptin transport. Inhibition of leptin could have had survival advantages during starvation. Many feeding substances have evolved affecting neurogenesis, neuroprotection, and cognition [20]. Therefore, to avoid the danger of hypoglycaemia to the brain, insulin resistance might have been favorably selected during evolution [14]. To compensate for the metabolic cost of encephalization, lower mass-specific metabolic rates in other tissues like the gut were favored [17]. Alternatively, the high consumption of protein and low carbohydrate (particularly the past 2 million years “The Ice Age”) favored the selection of insulin resistance, which resulted in increased hepatic gluconeogenesis and decreased peripheral glucose uptake.

Insulin resistance is a state in which insulin is incapable of exerting its biological effects lowering plasma glucose levels. It is a link between MS, glucose intolerance, hypertension, and dyslipidemia and is therefore related to cardiovascular disease. Hyperinsulinemia, resulting as a consequence of insulin resistance, leads to obesity due to the lack of the anabolic effects of this hormone upon lipid metabolism; lipogenesis is increased in adipocytes, lipid catabolism decreases, thermogenesis is lowered, and muscle mitochondrial oxidative capacity is increased [24]. Human ancestors commonly faced periods of feast followed by periods of famine. This second strategy preserved brain function and fetal/placental/mammary tissue functioning during reproduction [17, 25–27].

With intellectual progress, human ancestors migrated out of Africa and found fertile grounds near rivers that enabled the natural growth of wild crops (cereals and legumes) where four of the most important domesticated animals (cows, goats, sheep, and pigs) could be found, allowing the development of agriculture and farming. With domestication, animal diet changed from grazed to feed grain (rich in fatty acids omega-6) [14, 16] and increased fat deposits under the skin, within the abdomen, between and within muscles [14]. Fatter animals served as food for humans. Dairy products can be high in saturated fats which increase coronary artery disease. In villages, women could make clothes to protect themselves from the cold weather, leaving aside adaptive thermogenesis [14]. An increase in fat consumption therefore led to obesity and its cardiovascular consequences.

The advent of the industrial revolution, increased cereal production, and the composition of the diet significantly changed. Highly processed cereals were rich in carbohydrates, and omega-6 fatty acids, but low in omega-3 fatty acids and antioxidants in comparison with leafy green vegetables [16]. Refined sugars began to be consumed in large scale in processed food and beverages that resulted in significant postprandial hyperinsulinemia exposing the disadvantages of the insulin resistant genotype [28]. The sodium consumption was dramatically increased, while potassium, complex carbohydrates and fiber were substantially decreased; saturated fatty acids, omega-6, and trans-fatty acids replaced the unsaturated fatty acids and omega-3 [16]. The increase in sodium consumption increased water retention by the body and therefore favored hypertension. The consumption of micronutrients decreased, while the calorie intake increased [14, 15].

**Figure 2:**
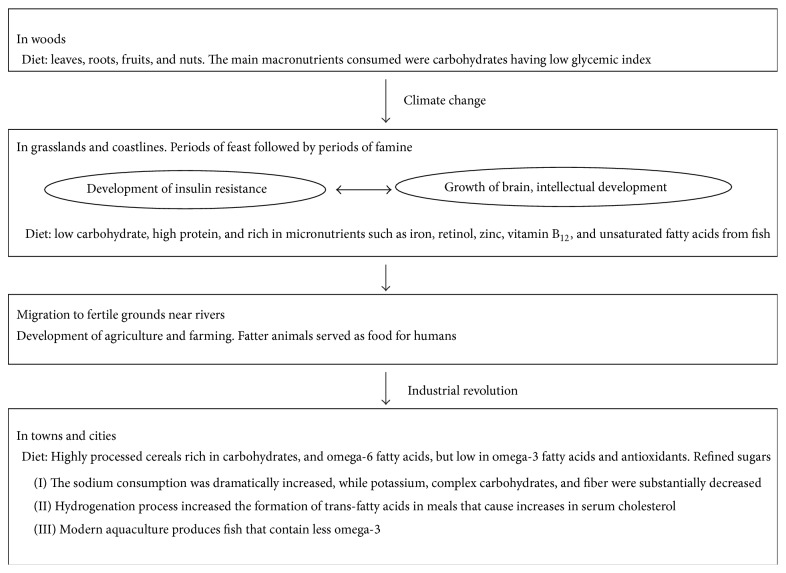
Major changes in human nutrition during evolution.

During the industrial revolution, the screw press and solvent extraction processes made the extraction of oil from seeds and its hydrogenation possible. The hydrogenation process increased the formation of trans-fatty acids in meals that cause increases in serum cholesterol which is related to atherosclerosis. Modern aquaculture produces fish that contain less omega-3. The composition of chicken egg has high concentrations of omega-6 to omega-3 ratio 19 : 9, while milk and cheese of animals fed with grains lack eicosapentaenoic (EPA), docosahexaenoic (DHA), and arachidonic (AA) acids. The increased consumption of fatty acids omega-6 and the decrease of omega-3 increased the eicosanoid metabolic products from AA, such as prostaglandins, thromboxanes, leucotrienes, hydroxy fatty acids, and lipoxins that contribute to the formation of thrombus and atheromas and to inflammatory disorders and insulin resistance [16]. Major changes in nutrition during human evolution are illustrated in Figure 2.

## 3. Selection of a Thrifty Genotype

The thrifty or diabetic genotype theory was proposed by Neel (1962) [26], based on epidemiological studies in northwest America, where there is a high incidence of type 2 diabetes. The theory proposes that “economic or diabetic genes” were selected during evolution when food was scarce and have been inherited to this day. These genes allowed individuals to store excess food as fat when it was available and then benefit from these stores during long periods of famine. Evolutionary medicine suggests that diseases are the result of an incompatibility between human evolutionary design and the current style of human life. Genes were selected to adapt human beings to their ancestral nutrition style and rate of activity over millions of years [29]. Today, environmental conditions have changed and humans face new conditions with an evolutionary design adapted to previous conditions that leads to obesity and diabetes. The study of the nutrition of human ancestors throughout the evolution described in the previous section may help us to understand the role played by insulin and insulin resistance [25, 26] in survival and the importance of the selection of a thrifty genotype; however, these genes may predispose to diseases in an environment with abundant sources of food rich in carbohydrates [27].

As an example of more recent gene selection to ancestral conditions, the presence of a functional variant of the A1 gene ATP-binding cassette transporter (ABCA1) is unique to populations of Native Americans and their descendants. This gene is determinant for low levels of HDL-C and may have contributed to the evolution of adaptation of Native American [30, 31] populations. However, nowadays the presence of this transporter leads to a higher incidence of diabetes, multiple sclerosis, and obesity. This variant of the gene may also confer protection against certain infectious and/or thrombotic disorders [32].

Another recent example can be found in Prehispanic Mesoamerica where the main source of energy was cereal instead of meat. 75% of the calories consumed by the Mayans came from maize. The use of maize as the dietary base of these civilizations and adoption of agricultural methods in Mesoamerica might have acted as possible forces influencing natural selection. Farmers gradually changed their active life to a sedentary life and their genotypes became maladaptive to their lifestyle [33]. Therefore, with the adoption of sedentarism, health deteriorated.

The theory of the thrifty genotype has been extended to scarce food during early stages of development and stressing conditions leading, through epigenetic changes, to low weight at birth that results in higher incidences of metabolic and cardiovascular diseases during adult life [34, 35]. Neonates with slow growth have less subcutaneous fat stores but similar intra-abdominal fat to normal-weight neonates [36]. Low weight favors energy allocation to specific tissues or fuel depots. The organism can therefore adjust the distribution of nutrients for the maintenance of specific tissues adapting to adverse conditions. The life history theory explains the trade-offs in the distribution of nutrients to tissues and natural selection determines the best strategy [37]. Age, gender, body size, growth rate, current energy stores, and reproductive status constitute determining factors influencing the strategy for the best distribution of energy.

One of the advantages of obesity resulting from the acquisition of a thrifty genotype was cerebral growth and female reproductive fitness. The reproductive and child rearing processes depend heavily on the accumulation of adipose tissue. Sexual selection acted upon adipose tissue distribution and contributed to reproductive fitness by altering the opportunities to mate, leading to the selection of individuals bearing this trait. It also contributes to the mechanisms that regulate female reproductive function [38]. Leptin acts on the availability of metabolic fuels in female reproduction to supply energy to the offspring [39, 40]. Adipose tissue also supplies us with the energy needed for the development of the human brain early in life, when this organ contributes importantly to body weight [41]. It acts as a reserve in the newborn and infant to face growth [38, 42].

An alternative theory to the thrifty genotype hypothesis has been proposed. It states that genetic drift rather than positive selection could have been a dominant factor for the evolution of obesity [43, 44]. According to this theory, starvation periods would have caused great mortality and it concludes that if only the individuals carrying the thrifty genotype were selected, nowadays everyone would be obese. It explains that the thrifty genotype genes would have disappeared since obesity might have been selected against by the risk of predation and by the sporadic occurrence of famine in current times. It proposes that, later on, the risk of predation was diminished because of the development of social behavior, the invention of weapons, and the discovery of fire and the distribution of body fat gradually changed favoring obesity due to mutations and drift. This could be the explanation of why nowadays most people are not obese [43].

Obesity is also associated with changes in the intestinal microbiota during evolution. The internal microbiome has adapted to diet changes and coevolution has played an important role in the relation between the host and the pathogen. Our bodies provide habitat and nutrition for organisms which improve our physical condition by metabolizing food which our digestive enzymes can not break and that constitute the human microbiota. These organisms contribute to 10% of the energy required by the human bodies. Our microbiota also protects against harmful microorganisms and participates in the development of the immune and gastrointestinal systems. In obese people, the intestinal microbiota favors the accumulation of fat since it includes bacteria that are able to metabolize ingested carbohydrate and provide additional energy that contributes to fat accumulation [45]. Thus, the intestinal microbiota promotes weight gain and fat accumulation, promoting low-grade inflammation and increasing cardiovascular risk. Lipopolysaccharides (LPS) of the outer surface of Gram-negative bacteria of the microbiota are exposed to the body and generate low-grade inflammation, insulin resistance, and increased cardiovascular risk and are involved in the rupture of the atherosclerotic plaque as part of metabolic endotoxemia [46].

## 4. Selection of an Enhanced Inflammatory Function

Our ancestors fought commensal organisms, pests that threatened their life, and chronic parasitic infestation and therefore were exposed to a greater infectious load than we currently face. During evolution, there was a selection of individuals with large portions of the genome dedicated to inflammatory responses and innate immunity to face infections and allow survival after trauma [38]. Adipose tissue resulting from a thrifty genotype also provides the necessary energy for the high cost of the immune system [47]. The contribution of energy reserves for immune function is evidenced by the reduced survival of low relative weight patients [42]. A high metabolism is needed to counteract infections with the need to synthesize immunoglobulins and acute phase proteins and other processes such as inflammation and fever [48]. To address these infectious challenges, a lipolytic response that releases energy from adipose tissue is triggered [49].

The adipose tissue has been considered as a nonhealthy tissue, but it may be more appropriately considered as an activator of the immune system helping in the protection against infectious diseases. Therefore, different infectious loads may have acted as evolutionary pressures since immunity is an important function of adipose tissue during malnutrition periods [38]. Active immunity could have been the common ancestor of type 2 diabetes and atherosclerosis. Infections are involved in the development of atherosclerosis, and when multiple pathogens are present the risk of a cardiovascular event is increased [50].

Adaptive immunity is restricted by the shortening of telomeres as an energy saving mechanism since telomere length is mainly limited by its energy cost. Therefore, shorter telomeres must accompany a thrifty phenotype. A less developed immune system favors chronic infections, stress from inflammatory processes, premature aging, and death. With a longer reproductive life, fitness costs of premature aging are higher [51].

Plaque formation in atherosclerosis is linked to microorganisms which form complexes of circulating toxins, while lipoproteins bind and inactivate microorganisms. Such complexes clog arteries causing ischemia and cell death in the arterial wall and cause vulnerable plaques [52]. Accompanying the rise of chronic diseases caused by our proinflammatory design, the appearance of some allergic and autoimmune diseases has increased during evolution. The increase in the prevalence of these diseases might be due to the reduction of exposure to pathogens, as explained by the “hygiene hypothesis.” These diseases are now listed as man-made [53].

## 5. Inflammation and Insulin Resistance

Insulin resistance is associated with a thrifty and proinflammatory human genotype and design. Proinflammatory molecules produced by adipose tissue are involved as active participants in the development of insulin resistance and increased risk of cardiovascular disease associated with obesity [54–56]. FFAs produce low-grade inflammation in skeletal muscle and liver. Therefore, high FFA levels (due to obesity or high-fat meal) participate in the development of insulin resistance in skeletal muscle and liver, producing low-grade inflammation, which contributes to the development of atherosclerotic vascular disease [57]. A key signal for immunity/inflammation may be leptin [58].

Insulin resistance is characterized by excessive generation of potentially cytotoxic ROS concentrations in endothelial cells [59]. Reactive oxygen or nitrogen species play an important role in myocardial damage and repair. Oxidative stress is increased in hyperglycemia, hyperlipidemia, and insulin resistance and in the diabetic myocardium and diminishes adaptability to ischemia-reperfusion. Impaired glucose flow, mitochondrial disorders, and decoupling nitric oxide synthase in the presence of a decrease in antioxidant defense and deterioration of cellular prosurvival signaling may render the myocardium more vulnerable to injury, diabetic remodeling, and cardiac failure [60].

NADPH oxidases (NOX) are key enzymes in insulin resistance and vascular dysfunction mediated by oxidative stress. There are isoforms of these enzymes that generate superoxide which promotes insulin resistance [59]. This finding provides a biochemical explanation for fatty liver, atherogenic dyslipoproteinemia, and hyperglycemia, thus explaining accelerated atherosclerosis and disease in small vessels [61]. Interestingly, it is known that some of the NOX isoforms are present in bacteria, indicating that these isoforms appeared early in evolution [62].

## 6. Inflammatory Function, Cardiovascular Disease, and MS

The greater susceptibility of individuals to develop cardiovascular disease and MS is related to the human thrifty genotype and proinflammatory design. Patients with atherosclerosis and MS have an altered chronic inflammatory function. Markers of inflammation and innate immune responses, including C-reactive protein (CRP), IL-6, TNF-*α*, and several cell adhesion molecules, are linked to the occurrence of myocardial infarction and stroke among patients with coronary heart disease [3]. The generation of endothelial adhesion molecules, proteases, and other mediators that enter the circulation in soluble form is mediated by inflammatory cytokines involved in vascular inflammation [63, 64]. Adipose tissue generates inflammatory mediators associated with atherothrombosis. The use of inflammatory biomarkers for detecting the risk of atherosclerosis and other cardiovascular and metabolic disorders has increased and their detection is becoming more widespread complementing the use of plasma lipid profiles [65, 66]. In cardiovascular events, central obesity represents a significant risk factor. An increased activation and a reduced sensitivity to the physiological and pharmacological antiaggregating agents of platelets also play a major role in the increased cardiovascular risk [67].

## 7. Inflammation and Hypertension

The human proinflammatory design and a design adapted to low salt ingestion favor the development of hypertension when humans are faced with the nowadays improved hygiene environment and with the modern intake of salt. Both acute and chronic inflammation processes are accompanied by changes in water content in the body and therefore in blood pressure. Acute inflammatory episodes cause local and systemic water loss and sometimes reductions in blood pressure as part of a slow inflammatory process. Loss of water by perspiration is high during acute inflammatory lapses. Additionally, an adequate adaptive immune response with proliferation of B and T cells and proliferation of neutrophils and monocytes needs enormous amounts of water. Water is actively retained to counteract systemic water loss during acute inflammation [68].

In contrast, during chronic inflammation, there is increased water retention in the inflamed tissue and water is reabsorbed in the kidney. This leads to a net increase of the body water volume which might underlie essential hypertension. Water retention hormones, such as angiotensin II have proinflammatory activities [68]. Hypertensive stimuli such as angiotensin II and salt cause elevation in pressure, through central stimulation, inducing direct effects on the kidney and the vasculature. New antigens are formed that promote the activation of T cells that move to the kidney and the vasculature and emit signals that promote the entrance of macrophages. These inflammatory cells release cytokines that cause vasoconstriction and promote sodium and water retention, causing severe hypertension [21].

For millions of years, the ancestors of humans were adapted to an environment free of salt, with an elevated evolutionary pressure for the selection of salt conserving genes such as the angiotensinogen gene [69] and the ACE gene [22]. Therefore, hypertension also has an evolutionary origin based on salt intake. There are variants of salt conserving enzymes whose activity is associated with an increase in water and sodium retention. These variants may have had some advantages for humans to adapt to the ancestral environment before the human race expanded to Asia and Europe. However, when humans inhabited regions where the climate became colder or wetter, salt and water retention became harmful. The presence of these genes increased susceptibility to hypertension [70]. The change of salt ingestion of the human species has represented an evolutionary mismatch with terrible health consequences [22]. Excessive salt increases water retention by peripheral tissues and in the kidney, increasing blood pressure.

Hypertension could also be caused by maladaptation to bipedalism [23]. One of the most accepted theories of the appearance of bipedalism states that it is a possible consequence of ancestral men trying to obtain food by fishing in shores near shallow water [71] and, an inability of the autonomous nervous system to cope continually with this situation throughout life, may contribute to the current epidemic of hypertension [23].

## 8. Toll-Like Receptors Link Inflammation to Cardiovascular Diseases

For evolutionary biologists and ecologists, the understanding of the evolution of the immune system has been a challenge as they are trying to associate the natural selection process with the infectious diseases. Both innate immunity and adaptive immunity may contribute to the pathophysiology of hypertension and metabolic and cardiovascular diseases. Toll-like receptors (TLRs) could provide the link between the human proinflammatory design and development of metabolic and cardiovascular diseases. Toll-like receptors (TLRs) are responsible for the identification of bacteria, fungi, protozoa, and viruses. They represent recognition mechanisms evolutionarily conserved that are related to microbial pathogen patterns and constitute the starting point of the inflammatory responses [72]. According to the ligands they target, two subclasses of TLRs have been described in vertebrates [73].

The evolutionary origin of the TLR signaling pathway is still unknown. It is highly conserved from insects to vertebrates, with an increase in its components and in the complexity of the signaling pathway. These changes seem to be associated with alterations in the living environment. The TLR signaling pathway might even be present in a common ancestor of sponges and eumetazoa with gene duplication, changes in the adaptive molecular structure of extended genes, and conservation of the motif of the NF-*κ*B gene [74].

TLRs have also been identified in humans, as functional receptors both in the plasma membrane and in the intracellular space, mainly in vascular and immune cells, myocytes, and platelets. Interestingly, the TLRs interact with endogenous ligands that are also elevated in diabetes and cardiovascular diseases such as FFA, oxidized LDL (Ox LDL), HSP (heat shock protein) 60 and HSP 70, fibronectin, and fibrinogen [74].

TLR4 protein expression have been implicated in the development and progression of cardiovascular diseases, since their levels increase with hypertension [75]. They have also been associated with initiation and progression of atherosclerosis. Recent findings have shown increased expression of TLR2/TLR4 signaling ligands, and functional activation in diabetic subjects [76]. An upregulation of TLR4 was observed after myocardial infarction in the heart of mice.

In adipocytes, a deficit of TLR4 uncouples the inflammatory signaling during and after ingestion of a high-fat diet. This deficit is also involved in insulin resistance and in muscle and glucose intolerance. Insulin resistance in adipose tissue and diet-induced obesity is not present when a functional mutation in TLR4 and TLR2 occurs [77].

TLRs are involved in the pathogenesis and progression of various liver diseases. In the liver, blood from the gastrointestinal tract, enriched with nutrients and antigens, passes through the sinusoids where it is in close contact to antigen-presenting cells and lymphocytes, thus functioning as an immunological organ [78]. The main hepatic complications of obesity and metabolic diseases are related to inflammatory hepatic steatosis and steatohepatitis. Fatty liver disease is characterized by infiltration of fat and excessive accumulation of lipids such as triglycerides. The levels of expression of inflammatory mediators in the liver are altered in obese patients even if histopathological manifestations are not visible. The liver from patients with nonalcoholic fatty liver disease (NAFLD) seems to respond better to the pathways of TLR activators [78]. NAFLD is associated with increased risk of coronary heart disease, heart abnormalities, left ventricular dysfunction, hypertrophy, heart failure, valvular heart disease, and arrhythmias. It also alters systemic/hepatic insulin resistance, causing atherogenic dyslipidemia and the release of a variety of proinflammatory mediators, profibrogenic and procoagulant molecules that are important in the pathophysiology of cardiac complications [79].

## 9. Conclusion

Our ancestors faced a greater infectious load and selection pressures for proinflammatory genes that helped them survive were present. They also faced periods of famine that pushed for a thrifty genotype. Both features depended on the ability to accumulate fat reserves. Similarly, immune senescence has increased with a longer lifespan in the present populations. Our current lifestyle has no need for these genotypes and/or these ancestral genes. Nevertheless, they are still present and as long as there are no significant changes in our lifestyle to keep them at bay, we will be subjected to the consequences of their activation. The advantages of high cytokine response and a moderate insulin resistance in the past exceeded the potential harmful effect of atherosclerosis. However, in our current environment, these effects can be the basis for our current epidemic of obesity and cardiovascular disease. From the evolutionary point of view discussed in this paper, it seems that the evolution of the forms for nutrition has exceeded the early human programming, putting the species at risk for metabolic and cardiovascular diseases. If the combination of the human biological design and the current environment is no longer compatible and the species has developed a higher risk for diabetes and cardiovascular disease, prevention may be the most recommended action. Prevention measures should include modifying our current lifestyle to a style of life closer to that of our ancestors. Prevention should start very early, even in utero and during early stages of life, due to the establishment of epigenetic programs that lead to predisposition to diseases in adults. It should also be aimed primarily at people with hereditary predisposition.

## Figures and Tables

**Table 1:** Actual conditions to which humans are not well adapted and disease to which we are at a higher risk of developing due to current environmental conditions.

Condition to which humans are not well adapted	Environmental conditions to which humans adapted	Risk derived from poor adaptation to conditions	Reference
Low-grade chronic inflammation	Great infectious burden with an active immune system	Low-grade systemic inflammation that leads to metabolic syndrome, diabetes, cardiovascular diseases, and hypertension	[1, 21]

Abundant diet high in carbohydrates	Famine and necessity to store fat	Increased risk of obesity, insulin resistance, metabolic syndrome, and type 2 diabetes	[5]

Sedentarism	High physical activity	Insulin resistance, obesity, metabolic syndrome, and type 2 diabetes	[1]

Diet high in salt	Little salt in the diet	Hypertension	[22]

Bipedalism	Quadruped movement	Orthostatic intolerance and hypertension	[23]

Longevity	Shorter lifespan	Increased exposure to the abovementioned risk factors	[12]

## Abreviations

ACE: Angiotensin converting enzyme
AA: Arachidonic acid
ABCA1: ATP-binding cassette transporter A1 gene
DHA: Docosahexaenoic acid
EPA: Eicosapentaenoic acid
FFA: Free fatty acid
HDL: High density lipoprotein cholesterol
HPA: Hypothalamus-pituitary-adrenal axis
LPS: Lipopolysaccharides
LDL: Low-density lipoprotein
MS: Metabolic syndrome
NOX: NADPH oxidase
NAFLD: Nonalcoholic fatty liver disease
ROS: Reactive oxygen species
TLRs: Toll-like receptors.

## References

[1] Ridker P. M. (2002). On evolutionary biology, inflammation, infection, and the causes of atherosclerosis. *Circulation*.

[2] Wells J. C. K. (2009). Ethnic variability in adiposity and cardiovascular risk: the variable disease selection hypothesis. *International Journal of Epidemiology*.

[3] Ridker P. M., Hennekens C. H., Buring J. E., Rifai N. (2000). C-reactive protein and other markers of inflammation in the prediction of cardiovascular disease in women. *The New England Journal of Medicine*.

[4] Fernández-Real J.-M., Ricart W. (1999). Insulin resistance and inflammation in an evolutionary perspective: the contribution of cytokine genotype/phenotype to thriftiness. *Diabetologia*.

[5] Pickup J. C. (2004). Inflammation and activated innate immunity in the pathogenesis of type 2 diabetes. *Diabetes Care*.

[6] Stern M. P. (1995). Diabetes and cardiovascular disease: the common soil hypothesis. *Diabetes*.

[7] Yudkin J. S., Stehouwer C. D. A., Emeis J. J., Coppack S. W. (1999). C-reactive protein in healthy subjects: associations with obesity, insulin resistance, and endothelial dysfunction: a potential role for cytokines originating from adipose tissue?. *Arteriosclerosis, Thrombosis, and Vascular Biology*.

[8] Hak A. E., Stehouwer C. D. A., Bots M. L. (1999). Associations of C-reactive protein with measures of obesity, insulin resistance, and subclinical atherosclerosis in healthy, middle-aged women. *Arteriosclerosis, Thrombosis, and Vascular Biology*.

[9] Festa A., D'Agostino R., Howard G., Mykkänen L., Tracy R. P., Haffner S. M. (2000). Chronic subclinical inflammation as part of the insulin resistance syndrome: the insulin resistance atherosclerosis study (IRAS). *Circulation*.

[10] Kobayasi R., Akamine E. H., Davel A. P., Rodrigues M. A. M., Carvalho C. R. O., Rossoni L. V. (2010). Oxidative stress and inflammatory mediators contribute to endothelial dysfunction in high-fat diet-induced obesity in mice. *Journal of Hypertension*.

[11] Da Silva J. A. P. (1999). Sex hormones and glucocorticoids: interactions with the immune system. *Annals of the New York Academy of Sciences*.

[12] Bauer M. E. (2005). Stress, glucocorticoids and ageing of the immune system. *Stress*.

[13] Franceschi C., Capri M., Monti D. (2007). Inflammaging and anti-inflammaging: a systemic perspective on aging and longevity emerged from studies in humans. *Mechanisms of Ageing and Development*.

[14] Pijl H. (2011). Obesity: evolution of a symptom of Affuence. How food has shaped our existence. *Netherlands Journal of Medicine*.

[15] Milton K. (2000). Back to basics: why foods of wild primates have relevance for modern human health. *Nutrition*.

[16] Simopoulos A. P. (2001). Evolutionary aspects of diet, essential fatty acids and cardiovascular disease. *European Heart Journal Supplements*.

[17] Mann N. (2000). Dietary lean red meat and human evolution. *European Journal of Nutrition*.

[18] Fehm H. L., Kern W., Peters A. (2006). The selfish brain: competition for energy resources. *Progress in Brain Research*.

[19] Guarner V., Alvarez-Buylla R. (1991). Changes in brain glucose retention produced by the stimulation of an insulin-sensitive reflexogenic zone in rats. *Journal of the Autonomic Nervous System*.

[20] Banks W. A. (2012). Role of the blood-brain barrier in the evolution of feeding and cognition. *Annals of the New York Academy of Sciences*.

[21] Harrison D. G., Guzik T. J., Lob H. E. (2011). Inflammation, immunity, and hypertension. *Hypertension*.

[22] Batuman V. (2012). Salt and hypertension: an evolutionary perspective. *Journal of Hypertension*.

[23] Sánchez-Torres G., Sanchez Torres G., Guarner V. (2012). Bipedalismo y el síndrome de intolerancia al ortostatismo. *Cardiología Evolutiva*.

[24] Mäkinen S., Skrobuk P., Nguyen Y. H., Koistinen H. (2013). Mechanisms of insulin resistance. *Duodecim*.

[25] Bogin B. (1998). From caveman cuisine to fast food: the evolution of human nutrition. *Growth Hormone and IGF Research*.

[26] Neel J. V. (1962). Diabetes mellitus: a ‘thrifty’ genotype rendered detrimental by ‘progress’?. *The American Journal of Human Genetics*.

[27] Hales C. N., Barker D. J. P. (1992). Type 2 (non-insulin-dependent) diabetes mellitus: the thrifty phenotype hypothesis. *Diabetologia*.

[28] Colagiuri S., Miller J. B. (2002). The ‘carnivore connection’—evolutionary aspects of insulin resistance. *The European Journal of Clinical Nutrition*.

[29] Amen-Ra N. (2006). Humans are evolutionarily adapted to caloric restriction resulting from ecologically dictated dietary deprivation imposed during the Plio-Pleistocene period. *Medical Hypotheses*.

[30] Aguilar-Salinas C. A., Canizales-Quinteros S., Rojas-Martínez R. (2009). Hypoalphalipoproteinemia in populations of Native American ancestry: an opportunity to assess the interaction of genes and the environment. *Current Opinion in Lipidology*.

[31] Acuña-Alonzo V., Flores-Dorantes T., Kruit J. K. (2010). A functional ABCA1 gene variant is associated with low HDL-cholesterol levels and shows evidence of positive selection in Native Americans. *Human Molecular Genetics*.

[32] Villarreal-Molina M. T., Aguilar-Salinas C. A., Rodríguez-Cruz M. (2007). The ATP-binding cassette transporter A1 R230C variant affects HDL cholesterol levels and BMI in the Mexican population: association with obesity and obesity-related comorbidities. *Diabetes*.

[33] Hünemeier T., Amorim C. E. G., Azevedo S. (2012). Evolutionary responses to a constructed niche: ancient mesoamericans as a model of gene-culture coevolution. *PLoS ONE*.

[34] Hattersley A. T., Tooke J. E. (1999). The fetal insulin hypothesis: an alternative explanation of the association of low birthweight with diabetes and vascular disease. *The Lancet*.

[35] Wells J. C. K. (2003). The thrifty phenotype hypothesis: thrifty offspring or thrifty mother?. *Journal of Theoretical Biology*.

[36] Harrington T. A. M., Thomas E. L., Frost G., Modi N., Bell J. D. (2004). Distribution of adipose tissue in the newborn. *Pediatric Research*.

[37] Stearns S. C., Nesse R. M., Haig D., Stearns S. C., Koella J. C. (2008). Introducing evolutionary thinking for medicine. *Evolution in Health and Disease*.

[38] Wells J. C. K. (2006). The evolution of human fatness and susceptibility to obesity: an ethological approach. *Biological Reviews of the Cambridge Philosophical Society*.

[39] Schneider J. E. (2004). Energy balance and reproduction. *Physiology & Behavior*.

[40] Kuzawa C. W. (1998). Adipose tissue in human infancy and childhood: an evolutionary perspective. *The American Journal of Physical Anthropology*.

[41] Norgan N. G. (1997). The beneficial effects of body fat and adipose tissue in humans. *International Journal of Obesity*.

[42] Lord G. (2002). Role of leptin in immunology. *Nutrition Reviews*.

[43] Speakman J. R. (2007). A nonadaptive scenario explaining the genetic predisposition to obesity: the ‘predation release’ hypothesis. *Cell Metabolism*.

[44] Speakman J. R. (2008). Thrifty genes for obesity, an attractive but flawed idea, and an alternative perspective: the ‘drifty gene’ hypothesis. *The International Journal of Obesity*.

[45] Kallus S. J., Brandt L. J. (2012). The intestinal microbiota and obesity. *Journal of Clinical Gastroenterology*.

[46] Manco M., Putignani L., Bottazzo G. F. (2010). Gut microbiota, lipopolysaccharides, and innate immunity in the pathogenesis of obesity and cardiovascular risk. *Endocrine Reviews*.

[47] Scrimshaw N. S., Schurch B., Scrimshaw N. S. (1990). Energy cost of communicable diseases in infancy and childhood. *Activity, Energy Expenditure and Energy Requirements of Infants and Children*.

[48] Beisel W. R. (1975). Metabolic response to infection. *Annual Review of Medicine*.

[49] Espinola-Klein C., Rupprecht H. J., Blankenberg S. (2002). Impact of infectious burden on extent and long-term prognosis of atherosclerosis. *Circulation*.

[50] Rook G. A. W. (2012). Hygiene hypothesis and autoimmune diseases. *Clinical Reviews in Allergy & Immunology*.

[51] Eisenberg D. T. A. (2011). An evolutionary review of human telomere biology: the thrifty telomere hypothesis and notes on potential adaptive paternal effects. *The American Journal of Human Biology*.

[52] Ravnskov U., McCully K. S. (2012). Infections may be causal in the pathogenesis of atherosclerosis. *The American Journal of the Medical Sciences*.

[53] Vendrell J., Broch M., Vilarrasa N. (2004). Resistin, adiponectin, ghrelin, leptin, and proinflammatory cytokines: relationships in obesity. *Obesity Research*.

[54] Beier J. I., Guo L., von Montfort C., Kaiser J. P., Joshi-Barve S., Arteel G. E. (2008). New role of resistin in lipopolysaccharide-induced liver damage in mice. *Journal of Pharmacology and Experimental Therapeutics*.

[55] Jager J., Grémeaux T., Cormont M., Le Marchand-Brustel Y., Tanti J.-F. (2007). Interleukin-1*β*-induced insulin resistance in adipocytes through down-regulation of insulin receptor substrate-1 expression. *Endocrinology*.

[56] Park E., Wong V., Guan X., Oprescu A. I., Giacca A. (2007). Salicylate prevents hepatic insulin resistance caused by short-term elevation of free fatty acids *in vivo*. *Journal of Endocrinology*.

[57] Boden G. (2006). Fatty acid—induced inflammation and insulin resistance in skeletal muscle and liver. *Current Diabetes Reports*.

[58] Demas G. E., Sakaria S. (2005). Leptin regulates energetic tradeoffs between body fat and humoural immunity. *Proceedings of the Royal Society B: Biological Sciences*.

[59] Sukumar P., Viswambharan H., Imrie H. (2013). Nox2 NADPH oxidase has a critical role in insulin resistance-related endothelial cell dysfunction. *Diabetes*.

[60] Ansley D. M., Wang B. (2013). Oxidative stress and myocardial injury in the diabetic heart. *Journal of Pathology*.

[61] Wu X., Chen K., Williams K. J. (2012). The role of pathway-selective insulin resistance and responsiveness in diabetic dyslipoproteinemia. *Current Opinion in Lipidology*.

[62] Zhang X., Krause K.-H., Xenarios I., Soldati T., Boeckmann B. (2013). Evolution of the Ferric Reductase Domain (FRD) Superfamily: modularity, functional diversification, and signature motifs. *PLoS ONE*.

[63] Malik I., Danesh J., Whincup P. (2001). Soluble adhesion molecules and prediction of coronary heart disease: a prospective study and meta-analysis. *The Lancet*.

[64] Packard R. R. S., Libby P. (2008). Inflammation in atherosclerosis: from vascular biology to biomarker discovery and risk prediction. *Clinical Chemistry*.

[65] Ross R. (1999). Atherosclerosis: an inflammatory disease. *The New England Journal of Medicine*.

[66] Van Gaal L. F., Mertens I. L., De Block C. E. (2006). Mechanisms linking obesity with cardiovascular disease. *Nature*.

[67] Anfossi G., Russo I., Trovati M. (2009). Platelet dysfunction in central obesity. *Nutrition, Metabolism and Cardiovascular Diseases*.

[68] Straub R. H. (2012). Evolutionary medicine and chronic inflammatory state—known and new concepts in pathophysiology. *Journal of Molecular Medicine*.

[69] Nakajima T., Wooding S., Sakagami T. (2004). Natural Selection and Population History in the Human Angiotensinogen Gene (AGT): 736 complete AGT sequences in chromosomes from around the world. *American Journal of Human Genetics*.

[70] Li X., Sun X., Jin L., Xue F. (2011). Worldwide spatial genetic structure of angiotensin-converting enzyme gene: a new evolutionary ecological evidence for the thrifty genotype hypothesis. *The European Journal of Human Genetics*.

[71] Niemitz C. (2010). The evolution of the upright posture and gait—a review and a new synthesis. *Naturwissenschaften*.

[72] Roach J. M., Racioppi L., Jones C. D., Masci A. M. (2013). Phylogeny of toll-like receptor signaling: adapting the innate response. *PLoS ONE*.

[73] Fornůsková A., Vinkler M., Pagès M. (2013). Contrasted evolutionary histories of two Toll-like receptors (Tlr4 and Tlr7) in wild rodents (MURINAE). *BMC Evolutionary Biology*.

[74] Song X., Jin P., Qin S., Chen L., Ma F. (2012). The evolution and origin of animal Toll-Like receptor signaling pathway revealed by network-level molecular evolutionary analyses. *PLoS ONE*.

[75] Bomfim G. F., Dos Santos R. A., Oliveira M. A. (2012). Toll-like receptor 4 contributes to blood pressure regulation and vascular contraction in spontaneously hypertensive rats. *Clinical Science*.

[76] Dasu M. R., Ramirez S., Isseroff R. R. (2012). Toll-like receptors and diabetes: a therapeutic perspective. *Clinical Science*.

[77] Bikman B. T., Summers S. A. (2011). Ceramides as modulators of cellular and whole-body metabolism. *The Journal of Clinical Investigation*.

[78] Broering R., Lu M., Schlaak J. F. (2011). Role of toll-like receptors in liver health and disease. *Clinical Science*.

[79] Ballestri S., Lonardo A., Bonapace S., Byrne C. D., Loria P., Targher G. (2014). Risk of cardiovascular, cardiac and arrhythmic complications in patients with non-alcoholic fatty liver disease. *World Journal of Gastroenterology*.

